# Nanopatterning via Self-Assembly of a Lamellar-Forming Polystyrene-*block*-Poly(dimethylsiloxane) Diblock Copolymer on Topographical Substrates Fabricated by Nanoimprint Lithography

**DOI:** 10.3390/nano8010032

**Published:** 2018-01-09

**Authors:** Dipu Borah, Cian Cummins, Sozaraj Rasappa, Ramsankar Senthamaraikannan, Mathieu Salaun, Marc Zelsmann, George Liontos, Konstantinos Ntetsikas, Apostolos Avgeropoulos, Michael A. Morris

**Affiliations:** 1 AMBER Centre & CRANN, Trinity College Dublin, College Green, Dublin, Ireland; cumminci@tcd.ie (C.C.); r_sola27@yahoo.co.in (S.R.); ramsankar.s@tcd.ie (R.S.); 2 Laboratoire des Technologies de la Microelectronique (CNRS), 38054 Grenoble, France; mathieu.salaun@cea.fr (M.S.); marc.zelsmann@cea.fr (M.Z.); 3 Department of Materials Science Engineering, University of Ioannina, University Campus-Dourouti, 45110 Ioannina, Greece; gliontos@cc.uoi.gr (G.L.); kntetsik@cc.uoi.gr (K.N.); aavger@cc.uoi.gr (A.A.)

**Keywords:** directed self-assembly, lamellar diblock copolymer, polyhedral oligomeric silsesquioxane (POSS), nanoimprint lithography, pattern transfer

## Abstract

The self-assembly of a lamellar-forming polystyrene-*block*-poly(dimethylsiloxane) (PS-*b*-PDMS) diblock copolymer (DBCP) was studied herein for surface nanopatterning. The DBCP was synthesized by sequential living anionic polymerization of styrene and hexamethylcyclotrisiloxane (D_3_). The number average molecular weight (*M*_n_), polydispersity index (*M*_w_/*M*_n_) and PS volume fraction (φ_ps_) of the DBCP were *M*_n_^PS^ = 23.0 kg mol^−1^, *M*_n_^PDMS^ = 15.0 kg mol^−1^, *M*_w_/*M*_n_ = 1.06 and φ_ps_ = 0.6. Thin films of the DBCP were cast and solvent annealed on topographically patterned polyhedral oligomeric silsesquioxane (POSS) substrates. The lamellae repeat distance or pitch (λ_L_) and the width of the PDMS features (*d*_L_) are ~35 nm and ~17 nm, respectively, as determined by SEM. The chemistry of the POSS substrates was tuned, and the effects on the self-assembly of the DBCP noted. The PDMS nanopatterns were used as etching mask in order to transfer the DBCP pattern to underlying silicon substrate by a complex plasma etch process yielding sub-15 nm silicon features.

## 1. Introduction

To maintain historic improvements in the performance of semiconductor electronics (Moore’s Law), the dimensions of critical circuit elements are shrinking towards 10 nm [1]. The most sophisticated semiconductor devices, such as microprocessors and memory chips, are patterned with high-resolution projection UV lithography [2]. However, sub-10 nm feature sizes are challenging and expensive to produce [3]. Alternative “top-down” techniques including thermal [4], e-beam [5,6] and X-ray [7] lithographies are largely unproven for manufacturing. Alternatively, the hierarchical self-assembly of molecular building blocks through molecular recognition and molecule-surface interactions [8] may have the potential for forming surface nanopatterns to create circuit elements. However, it is highly challenging to achieve the required long-range translational order and pattern robustness needed for manufacture with bottom-up approaches [9,10,11,12]. 

Long-range order of self-assembled systems can be achieved by directed self-assembly (DSA) where an external potential “guides” the self-assembling materials [13,14,15,16,17]. One form of guiding structure is well-defined surface topography (graphoepitaxy) and nanoimprint lithography (NIL) is a technique of simple and cost-effective means for generating topographical patterns [18,19,20], and can be used to generate substrate topography from a well-defined stamp [21]. Nanoimprinting can guide the self-assembly of a diblock copolymer (DBCP), controlling alignment (to surface direction) and orientation (relative to the surface plane), as was first reported by Li et al. on polystyrene-*b*-poly(methylmethacrylate) (PS-*b*-PMMA) [22]. The polystyrene-*b*-poly(dimethylsiloxane) (PS-*b*-PDMS) is a highly promising system and has been amenable to NIL based DSA [23,24]. PS-*b*-PDMS has a high Flory–Huggins parameter (*χ* = 68/*T* − 0.037) allowing formation of small microdomain sizes [25].

Work on the PS-*b*-PDMS system has been very largely limited to cylinder forming systems, and the lamellar forming compositions are expected to have a number of challenges, such as surface dewetting due to high hydrophobicity, and the formation of wetting layers [26]. Low PDMS surface energy and the possibility of forming bonds to surface silanol groups can lead to PDMS formation at the air and substrate interface [27], and for lamellar systems, this is likely to result in parallel orientation and 2D sheet-like structures. To enable proper graphoepitaxy, it is necessary to not only control sidewall chemistry, but also to produce appropriate surface chemistry to define pattern orientation.

In this work, we have attempted to define the required surface chemistry to demonstrate the practicality of using lamellar PS-*b*-PDMS systems. Polyhedral oligomeric silsesquioxane (POSS) materials can be tailored to allow definition of surface chemistry derivatives and three such POSS materials were studied here. It is shown that POSS can facilitate vertical orientation of lamellar. However, the neutral surfaces do not facilitate alignment of the patterns in graphoepitaxial structures, but can be used to understand surface chemistry effects in these systems.

## 2. Results and Discussion

### 2.1. DBCP Self-Assembly on PDMS-OH Brush Coated Substrate

**Scheme 1:**
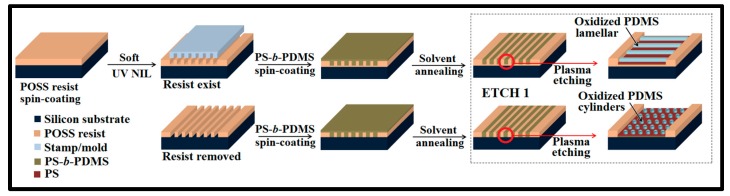
Schematic depicting the process flows of fabricating nanopatterned POSS template by the soft UV-NIL process, PS-*b*-PDMS self-assembly and the ETCH1 process. Two processes are shown, with and without residual resist removal.

**Figure 1:**
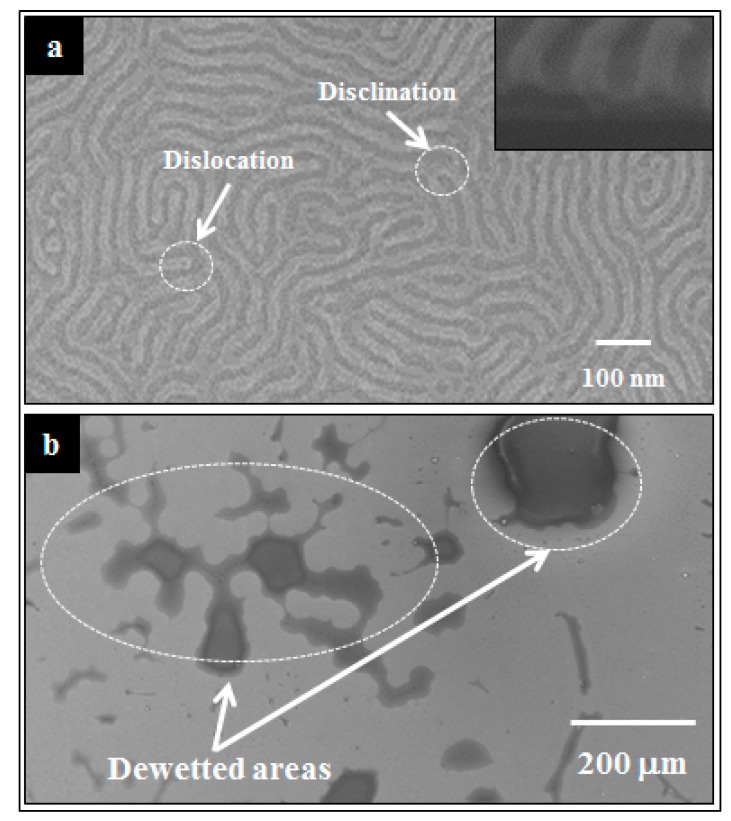
(**a**) Top-down SEM image (inset, cross-section image). Various defects noted in the micrograph and (**b**) top-down SEM images showing the coverage of microphase separated PS-*b*-PDMS film (as revealed by ETCH1 (CF_4_ and O_2_ etches)).

The details of the steps involved in the fabrication of POSS template and the subsequent DBCP self-assembly and plasma etching are presented in Scheme 1. The lamellar-forming PS-*b*-PDMS DBCP deposited on PDMS-OH brush (thickness ~4.3 nm as measured by ellipsometry) showed microphase separation after solvent annealing in toluene, as is evident from the SEM image of samples after ETCH1, Figure 1a. Note that no pattern was observed using Atomic Force Microscopy (AFM) studies of unetched, solvent-annealed films, suggesting that a PDMS wetting layer is formed at the air–polymer interface. The PDMS-OH brush favors PDMS (brush)–PDMS (DBCP) nteractions and a significantly lower PDMS surface energy, compared to PS results in a wetting PDMS layer at the substrate–polymer interface and at the polymer–air interface [24,26,27]. Note, however, that the SEM image shown in Figure 1a is not representative of the whole surface, since the DBCP dewets (Figure 1b) during solvent annealing (good coverage is observed after film casting). The films at the brush layers are generally poor, exhibiting multilayer pattern formation, low lamellar correlation lengths and defects such as disclinations, dislocations, etc. The SEM determined lamellae repeat distance or pitch (λ_L_), and the PDMS lamellae width (*d*_L_) are ~35 nm and ~17 nm. 

### 2.2. Effect of POSS Type on DBCP Self-Assembly on Planar POSS Coated Substrates

**Scheme 2:**
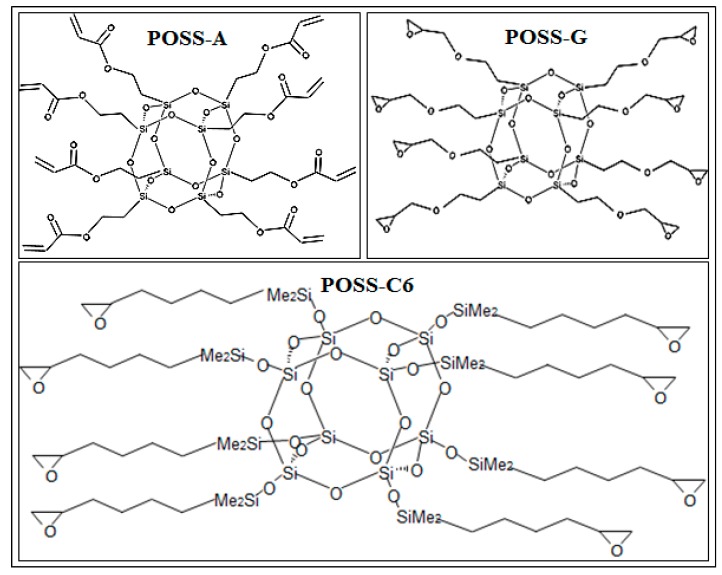
Structural details of the POSS resists used in the present study.

In an effort to improve the DBCP patterns, POSS materials were developed as an improved alternative to polymer brush. The surface properties of the POSS films can be controlled by grafting different ligands to the POSS cages, as is revealed from the data compiled in Table 1 for POSS-A, POSS-G, and POSS-C6, where the POSS cage is functionalized with eight aliphatic acrylo, glycidyl, and epoxide ligands, respectively. The structural formulae of the POSS derivatives are shown in Scheme 2. As seen in Table 1, POSS-A is the material of highest hydrophilicity, whilst POSS-C6 is the most hydrophobic. It is worthy to make a comparison of the surface free energies of POSSs with that of PS and PDMS. The surface free energies of PS and PDMS are 29.9 and 19.8 mNm^−1^, respectively [28,29]. It is evident that the surface free energies of POSS derivatives are higher than both PS and PDMS. It is understood that a polymer brush made of the minority block (PDMS here) that preferentially wets the substrate, is likely to have little effect on ordering of the DBCP [30]. Apparently, the POSSs substrates with higher surface free energy likely to provide the perfect energy barrier for the diffusion of PS-*b*-PDMS DBCP into the resist surface.

**Figure 2:**
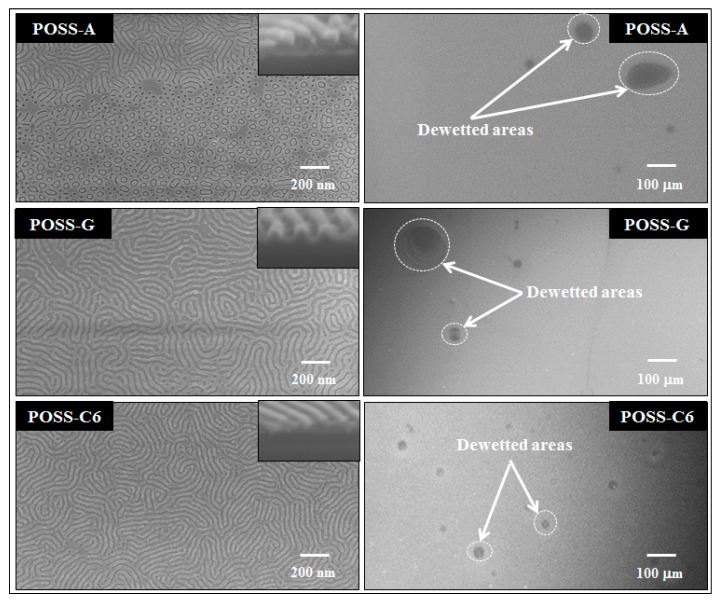
Top-down SEM images of lamellar PS-*b*-PDMS patterns (**left side**) (inset, cross-section images) and coverages of microphase separated PS-*b*-PDMS films (**right side**) following ETCH1 at silicon substrates modified with various POSS thin films.

Self-assembly of the PS-*b*-PDMS DBCP was followed on POSS modified planar substrates, and illustrative data presented in Figure 2. The surface coverage was much higher than on brush modified surfaces, with greater than 80% coverage being obtained in all cases (Figure 2). As well as the coverage advantages, it should be noted that POSS deposition is significantly simpler than brush attachment procedures. Note, however, that POSSs being silicon based compounds, might have a tendency to wet the PDMS block of the DBCP, forming a PDMS wetting layer at the DBCP–POSS interface. However, it is very difficult to isolate the PDMS wetting layer from the POSS resist in the cross-section SEM images (inset, Figure 2), because of the similar contrast. Figure 2 shows that the DBCP forms lamellar patterns for POSS-G and POSS-C6, however, in POSS-A, the morphology is more complex, showing well-defined regions of lamellar patterns and more irregular arrangements of the domains. The less well-defined areas have domains of differing sizes and shapes, ranging from pseudospherical to stripes. There are regions reminiscent of the phase transition between the lamellar phase and cylindrical phase found in the hexagonal perforated lamellar (HPL) structure [31]. Since this polymer composition is 60:40 for PS/PDMS, respectively, it is close to the phase region between the lamellar and cylinder structures where the HPL and IA3 gyroid structures exist, and this may be a complex mixture of these phases. The reason that this complex phase is only seen on the POSS-A film may be attributed to the fact that the surface chemistry favors a PDMS wetting layer, which effectively decreases the content of PDMS in the interior of the DBCP film and moves the composition towards the hexagonal regions.

### 2.3. Effect of POSS Topography on DBCP Self-Assembly

**Figure 3:**
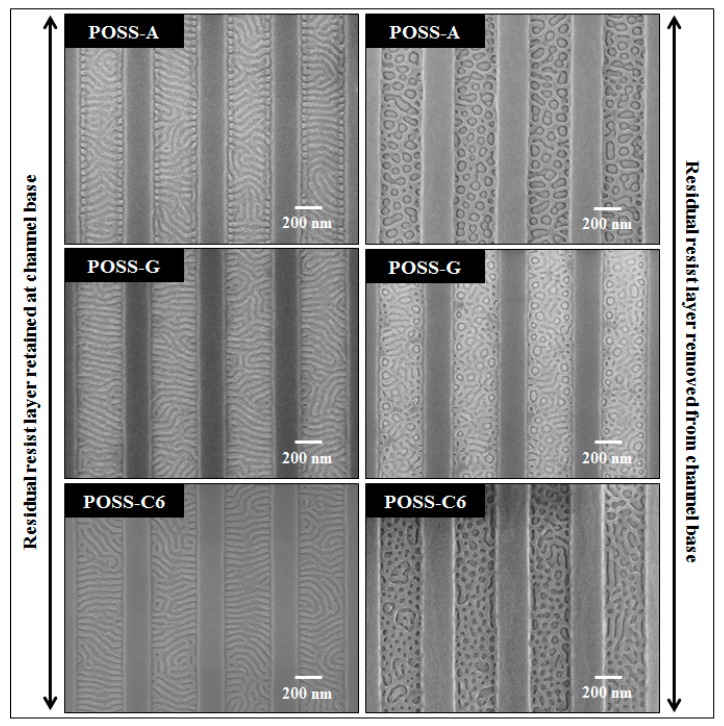
Top-down SEM images of lamellar-forming PS-*b*-PDMS at topographical POSS substrates before and after residual resist layer removal and following ETCH1.

The directed self-assembly (DSA) of PS-*b*-PDMS has been demonstrated for cylinder-forming PS-*b*-PDMS in several articles [26,32,33,34,35,36,37,38,39], however, there are only a few reports of lamellar-forming PS-*b*-PDMS systems [40,41]. In an effort to understand the effect of surface chemistries further, the lamellar-forming PS-*b*-PDMS DBCP films were deposited on NIL patterned POSS substrates. These topographical patterns are important because preferential interactions with blocks can be viewed in top down images. These NIL defined POSS topographies have been discussed elsewhere [24,32,42]. Briefly, NIL produces well defined channel or trench like structures. The channels were 50 nm deep, 270 nm wide, and the channel spacing was 500 nm. Also present is a 15 nm POSS resist layer residue. The deposition of DBCP was controlled using solution concentration and spin speed to just fill the channels so that the thickness is similar to the *L*_0_ of the DBCP, favoring vertical alignment of the domains. It should also be stated that the POSS structures are quite stable during the prolonged solvent anneal procedures when the DBCP is present (Figure 3). 

Preferential alignment of the lamellar to the channel side wall was not observed here. However, the topography allowed further understanding of the effects of surface chemistry changes. Figure 3 shows the results of PS-*b*-PDMS self-assembly on POSS topography in the presence and absence of the residual layer. With the resist layer present, all of the POSS structures provided lamellar arrangements. It is clear from the data presented in Figure 2 and Figure 3 that the POSS surfaces are essentially neutral, and do not favor either block. This neutrality is quite clearly seen in Figure 3, because the sidewalls favor an orthogonal arrangement of lamellar (i.e., across the trench), and both PS and PDMS lamellar exist at the sidewall. This is somewhat unexpected for POSS-A, since planar substrates yielded the HPL type arrangement. On this POSS, there is an obvious fine balance in energies that make both the HPL and lamellar structure approximately equally likely. However, the presence of topography makes the lamellar structure slightly more prevalent. When the POSS residual layer is removed, HPL type structures tend to dominate, suggesting the trench base is no longer neutral to both blocks, allowing the formation of a wetting layer and effective compositional changes in the interior of the POSS films. However, the sidewalls remain neutral, with both PS and PDMS present.

### 2.4. Pattern Transfer to Underlying Substrate for Nanopatterning

**Figure 4:**
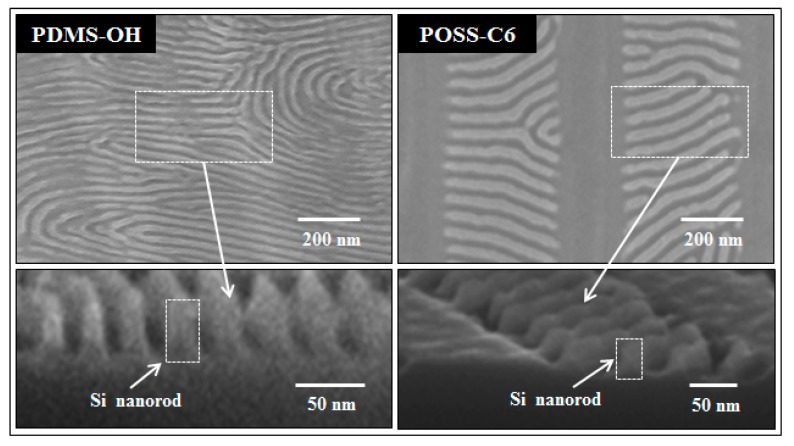
Top-down and high-resolution cross-section SEM images of PDMS patterns transferred to brush modified planar substrates and POSS-C6 patterned silicon substrates.

Pattern transfer both demonstrates the regularity of the polymer structure through the film (i.e., there is no complex 3D morphology) but also that the structure has practicality for fabrication of nanodimensioned substrate features. ETCH2 and ETCH3 were used to generate silicon features at both planar and topographical substrates. Figure 4 shows the top-down and cross-section SEM images of the silicon nanorod-type structures on a brush terminated planar silicon substrate and a POSS-C6 patterned silicon substrate. The pattern transfer is successful in both cases, and in all systems studied. Note that on the patterned substrates, the channel mesas have been largely removed, as they are clearly as etch resistant as the oxidized PDMS structures formed by ETCH1. The cross-section SEM image from the planar substrate shows that the silicon nanorod-like structure that is produced via this complex etch procedure has a feature size of ~14 nm, with a feature height of ~38 nm on the planar substrate. The nanoscale silicon features are slightly narrower in width than that of the initial oxidized PDMS features, due to a partly isotropic etch process. The PDMS patterns obtained on POSS-C6 patterned substrate without residual resist layer at the channel base upon pattern transfer, produced silicon nanorod-like features of thickness ~13 nm and a height of ~38 nm. 

## 3. Materials and Methods

### 3.1. Synthesis of Polymers and Molecular Characteristics

The lamellar-forming PS-*b*-PDMS DBCP was synthesized by sequential living anionic polymerization of styrene and hexamethylcyclotrisiloxane (D_3_), as described in the Supplementary Materials. The number average molecular weight (*M*_n_) and polydispersity index (*M*_w_/*M*_n_) of the DBCP were determined using size exclusion chromatography (SEC), and determined to be *M*_n_^PS^ = 23.0 kg mol^−1^, *M*_n_^PDMS^ = 15.0 kg mol^−1^, and *M*_w_/*M*_n_ = 1.06. The volume fraction (φ_ps_) of PS in the DBCP was determined by ^1^H-nuclear magnetic resonance (NMR) spectroscopy at 0.60. The hydroxyl-terminated PDMS (PDMS-OH) polymer brush was also synthesized via anionic polymerization techniques, and end-capped with 1–2 monomeric units of ethylene oxide (EO) in pyridine, and terminated with methanol (MeOH). M_n_ and *M*_w_/*M*_n_ were 5.5 kg mol^−1^ and 1.06, respectively. Full details of molecular characterization are available in Supporting Information.

### 3.2. POSS Materials and Synthesis of POSS-C6

OctaSilane POSS^®^ (POSS), Acrylo POSS^®^ (POSS-A) and Glycidyl POSS^®^ (POSS-G) were purchased from Hybrid Plastics (Hattiesburg, MS, USA). 1,2-epoxy-5-hexene, styrene, platinum(0)-1,3-divinyl-1,1,3,3-tetramethyldisiloxane complex solution (Karstedt´s catalyst), toluene and ethyl-l-lactate were purchased from Sigma Aldrich (Dublin, Ireland). The photoinitiators Irgacure^®^ 250 (iodonium(4-methylphenyl)[4-(2-methylpropyl)phenyl]-hexafluorophosphonate, 75 wt % in propylene carbonate), Irgacure^®^ 819 (bis(2,4,6-trimethylbenzoyl)-phenylphosphineoxide) and the sensitizer Genocure^®^ ITX (isopropyl thioxanthone) were generously provided by, respectively, BASF Resins (Berlin, Germany) and RAHN AG Energy Curing (Zurich, Switzerland). The epoxy-functionalized POSS monomer POSS-C6 was prepared according to methodology detailed previously [43]. OctaSilane POSS cages bearing eight dimethylsilyloxy groups were functionalized by a hydrosilylation reaction carried out on the Si–H functions. Typically, 2 g (1.96 mmol) of OctaSilane POSS were dissolved in 10 mL of anhydrous toluene. Subsequently, 1.96*8 mmol (8 ligands) of 1,2-epoxy-5-hexene (+10% excess) were added to the mixture with 2 drops of Karstedt’s catalyst. The reaction was carried out at 353 K during 3 h under argon. Solvent, catalyst, and material were removed under vacuum. Characterization details are available in Supporting Information.

### 3.3. Resist Preparation and Fabrication of POSS Templates by UV-NIL

Topographical substrates for graphoepitaxy experiments were fabricated using UV nanoimprint lithography and a PDMS elastomeric mold (so-called “soft UV-NIL”). POSS monomers were diluted in propylene glycol methyl ether acetate (PGMEA). Two moles (relative to epoxy groups) of Irgacure^®^ 250 photo-initiator and 0.5 wt % of Genocure^®^ ITX photo sensitizer were added to provide UV sensitivity. These solutions were spin-coated onto 4″ silicon (B doped, *p*-type, thickness 650 μm, and resistivity 6–14 ohm-cm) wafers of <100> orientation and a native oxide layer of ~2 nm, to form 50 nm thick resist films. Some of the POSS coated substrates were not patterned for planar surface studies. The PDMS mold (forming 50 nm deep, 270 nm wide trenches in the resist) was stamped into the resist layer at a pressure of 200 kPa, and the stack was then exposed to UV radiation (365 nm wavelength) for 3 min. Prior to imprinting, molds were treated with a fluorosilane anti-sticking compound (Optool DSX, Daikin Chemical, Dusseldorf, Germany) to allow even demolding. The resist residual layer at the bottom of the imprinted trenches (approx. 15 nm thick) could be removed by a CF_4_ (15 sccm) inductively coupled plasma (ICP) etch 4–8 s (varies with the resist type), which has been detailed before [21].

### 3.4. Deposition of PDMS-OH Brush on Silicon Substrates

Silicon substrates were ultrasonically degreased in acetone and IPA solutions for 5 min each, dried in flowing N_2_ gas, and baked for 2 min at 393 K in an ambient atmosphere, to remove any residual IPA. They were then cleaned in piranha solution (1:3 *v*/*v* 30% H_2_O_2_/H_2_SO_4_) at 363 K for 60 min, rinsed with deionized water, acetone, and ethanol before drying under N_2_ flow. Piranha activation removes contamination, and generates substrate surface hydroxyl groups. Hydroxyl-terminated PDMS brush solutions (1.0 wt % in toluene) were spin-coated (P6700 Series spin-coater, Specialty Coating Systems, Indianapolis, IN, USA) onto the wafers at 3000 rpm for 30 s. Samples were annealed in a vacuum oven (Townson & Mercer EV018, Wolflabs, York, UK) at 443 K under vacuum (1.3 kPa of residual pressure) for 6 h. This allows condensation of the brush hydroxyl end groups with surface silanol groups, resulting in polymer chain brushes being chemically attached to the substrate. Unbound polymers were removed by sonication (Cole-Palmer 8891 sonicator) and rinsing in toluene.

### 3.5. Deposition of PS-b-PDMS and Solvent Annealing

Thin films of PS-*b*-PDMS were prepared by depositing dilute solutions (e.g., 1.0 wt %) of the DBCP in toluene onto polymer brush anchored and unpatterned/patterned POSS substrates by spin coating (e.g., 3200 rpm and 30 s). As-cast thin films were solvent annealed in glass jars under saturated toluene environment at room temperature (~288 K) for 3 h. After removal solvent was allowed to evaporate at ambient conditions.

### 3.6. Plasma Etching of PS-b-PDMS Films and Pattern Transfer

All samples were plasma etched to allow easy imaging of the DBCP patterns and to transfer the polymer pattern to the substrate. Details of the etch processes are given elsewhere [33,34]. Briefly, an etch process (ETCH1) was used to convert the PS-*b*-PDMS pattern onto a silicon oxide-like pattern. A CF_4_ was used to remove any PDMS wetting layer at the air–polymer interface. An O_2_ (30 sccm) plasma etch was used to oxidize the PDMS into a silica-like structure. The steps used follow a similar methodology to that developed by Ross et al. [26]. The process removes the PS component and forms an oxidized form of PDMS on the substrate. These oxidized PDMS patterns were then used as an etch mask for pattern transfer (i.e., ETCH2). This etch consists of a CHF_3_/Ar plasma, and removes any non-oxidized PDMS from the surface. This process was followed by a selective silicon etch using CHF_3_ and SF_6_ to transfer the patterns into the underlying substrate. Any remaining polymer derived material was removed by ETCH3. The residual oxidized PDMS features were removed by a 10 s silica etch based on CHF_3_ and Ar. This was followed by an O_2_ etch to remove any residual PS and polymer brush. All etching was carried out in an OIPT Plasmalab System100 ICP180 (Oxford Instruments, Bristol, UK) etch tool. 

### 3.7. Material Characterization

Contact angles and surface free energy measurements were carried out using a Krüss DSA 100 (Krüss Optronic, Hamburg, Germany) goniometer. Contact angles were measured by the static sessile drop method and surface free energy was calculated from the measured contact angles of deionized water (DI), diiodomethane (DIM), and ethylene glycol (EG) using the Owens–Wendt model [44]. Film thickness (an average of five readings from different sample areas) was determined by ellipsometry (Plasmos SD2000 Ellipsometer, Filmetrics, Surrey, UK) at an incidence angle of 70°. A Varian IR660 (Agilent Technologies, Cheshire, UK) infrared spectrometer was used to record FTIR data. The measurements were performed in the spectral range of 4000–500 cm^−1^, with a resolution of 4 cm^−1^ and data averaged over 32 scans. Surface morphology and silicon nanostructures were investigated by scanning electron microscope (SEM) images, and were obtained by a high resolution (<1 nm) Field Emission Zeiss Ultra Plus-SEM (Carl Zeiss AG, Oberkochen, Germany) with a Gemini^®^ column operating at an accelerating voltage of 5 kV. The profile images of the surfaces were also used to measure the film thicknesses which agreed well with ellipsometer data.

## 4. Conclusions

We have demonstrated in this work the self-assembly and directed self-assembly (DSA) of a lamellar-forming PS-*b*-PDMS diblock copolymer (DBCP) system on substrates coated with PDMS-OH brush and POSS materials. The behavior of POSS materials in terms of wetting the DBCP domains can be obtained from DSA of the DBCP on patterned POSS substrates fabricated by soft UV nanoimprint lithography. The functionalization of the POSS materials have shown advantages of tuning the surface properties to improve the wetting property of the DBCP, direct self-assembly and pattern orientation/alignment. The lamellar DBCP forms oxidized PDMS patterns that can be successfully pattern transferred to underlying silicon to fabricate silicon nanoscale features with sub-15 nm feature size.

## Figures and Tables

**Table 1:** Contact angle (θ) and surface free energy (*SFE*) of POSS-A, POSS-G, and POSS-C6.

POSS Type	θ_DI_ (°)	θ_DIM_ (°)	θ_EG_ (°)	*SFE* (mN/m)
POSS-A	59.3	42.7	33.9	47.5
POSS-G	68.1	43.0	44.9	42.7
POSS-C6	83.6	57.3	65.1	31.1

## Supplementary Materials

The following are available online at http://www.mdpi.com/2079-4991/8/1/32/s1, Details of the synthesis and characterization of PS-*b*-PDMS DBCP, PDMS-OH polymer brush and characterization of synthesized POSS-C6.
