# Supplementary material for: Nanopatterning via Self-Assembly of a Lamellar-Forming Polystyrene-block-Poly(dimethylsiloxane) Diblock Copolymer on Topographical Substrates Fabricated by Nanoimprint Lithography
Source: Nanomaterials (Basel). 2018 Jan 9;8(1):32. doi: 10.3390/nano8010032 (PMC5791119; doi:10.3390/nano8010032)
Supplement: Supplementary file 1 [file nanomaterials-08-00032-s001.pdf]

Nanopatterning *via* Self-Assembly of a Lamellar-Forming Polystyrene-*block*-Poly(dimethylsiloxane) Diblock Copolymer on Topographical Substrates Fabricated by Nanoimprint Lithography

Dipu Borah,*[1] Cian Cummins,[1] Sozaraj Rasappa,[1] Ramsankar Senthamaraikannan,[1] Mathieu Salaun,[2] Marc Zelsmann,[2] George Liontos,[3] Konstantinos Ntetsikas,[3] Apostolos Avgeropoulos,[3] and Michael A. Morris*[1]

1. Synthesis and Characterization of PS-*b*-PDMS Diblock Copolymer (DBCP) and PDMS-OH Polymer Brush

1.1. Materials and Methods

1.1.1. Materials

Benzene (Panreac, 99.5%) was purified via distillation from CaH_2 and then stored in a high vacuum line on a cylinder, containing polystyrylithium, which incorporates a characteristic color, and indicates the purity of the benzene. Tetrahydrofuran (THF) (Fisher Scientific, 99.99%) was refluxed through metallic sodium, and then distilled through CaH_2 in Na/K alloy (3:1) under high vacuum conditions. Hexamethylcyclotrisiloxane (D_3) (Acros Organics, 98%) was put in a flask, diluted by an equal volume of purified benzene (C_6H_6), and stirred over CaH_2. The solution (D_3 and C_6H_6) was sublimed into a flask containing $PS^{(-)}Li^{(+)}$, through a short path distillation apparatus, where it was stirred in the presence of $PS^{(-)}Li^{(+)}$ for approximately 2 h at room temperature, sublimed again and finally stored at precalibrated ampoules. Styrene (Acros Organics, 99%) was purified by distillation from CaH_2 to dibutylmagnesium (Aldrich, 1 M solution in heptane), and then stored into precalibrated ampoules. *sec*-BuLi (Aldrich, 1.4 M in cyclohexane) was diluted in purified benzene, in a specific glass apparatus, under high vacuum techniques. Methanol (Fisher Scientific, 99.99%) was purified under high vacuum techniques through distillation from CaH_2 and freeze degassing into ampoules.

1.2. Synthesis of Polymers

For the synthesis of the polymers we employed anionic polymerization, via high vacuum techniques in specific glassware Pyrex apparatuses equipped with break seals for the two monomers, initiator, second solvent (THF), and terminating reagent (methanol). More details on the manipulations used are given elsewhere [1].

1.3. Synthesis of PS-b-PDMS DBCP

The polystyrene living chains were synthesized by polymerizing 7 g (0.067 mol) of styrene with *sec*-BuLi (0.128 mmol) in 100 mL of benzene at room temperature for 24 h. The solution exhibited a characteristic dark yellow to orange color, due to the polystyrene living ends. The number average molecular weight of the living chain was ~23,000 g/mol (molecular characterization results, Table S1). The appropriate amount of D_3 (7 g or 0.094 mol) was then added and left to react under stirring for 18 h until the color of the $PS^{(-)}Li^{(+)}$ disappeared. Then, an appropriate amount of THF was added to form a 1:1 solution of benzene/THF and left to react for 4 h at room temperature, until ~50% conversion of D_3 to PDMS occurred, as reported already in the literature [2]. Afterwards, in order to avoid and/or minimize the formation of back-biting reactions, which may lead to unwanted side products, the polymerization apparatus was left at -20 °C for approximately 7–10 days to achieve 100% conversion of D_3 to PDMS. The termination of the copolymer was performed by adding a small

amount (~1 mL) of MeOH. The number average molecular weight of the DBCP was calculated ~38,000 g/mol. More details are given elsewhere [2–4].

1.4. Synthesis of PDMS-OH Polymer Brush

The hydroxyl-terminated PDMS (PDMS-OH) polymer brush was synthesized by a living anionic polymerization of hexamethylcyclotrisiloxane (D_3) with *sec*-BuLi as initiator, end capped with 1–2 monomeric units of ethylene oxide (EO) in pyridine, and terminated with methanol (MeOH).

1.5. Instrumental Characterization

The equipment used for the characterization was a HT-GPC from Polymer Laboratories (PL-GPC-120) equipped with one pre-column for organic solutions, three PLgel 5μm MIXEDC columns and a refractive index (RI) detector. The solvent used was THF containing 0.5% pyridine at 35 °C with a flow rate of 1 ml/min. Size exclusion chromatography (SEC) was calibrated with eight PS standards (M_P: 4.300 g/mol to 3.000.000 g/mol) and always prior to calculating the polydispersity indices of the unknown materials a series of standard PS solutions were tested in order to examine the accuracy of the instrumentation.

Proton nuclear magnetic resonance (^{1}H-NMR) spectroscopy was used for determining the composition and the isomeric microstructures of the materials and was carried out in $CDCl_3$ at 30 °C using a Bruker AVANCE II spectrometer. Data were processed using UXNMR (Bruker) software.

Differential scanning calorimetry (DSC) measurements were accomplished by using a TA Instrument model Q20 with TA advantage software v5.2.6.

1.6. Results and Discussion

The molecular characterization results from size exclusion chromatography (SEC) and proton nuclear magnetic resonance spectroscopy (^{1}H NMR) for the DBCP are given in **Table S1 and Table S2** for PS-*b*-PDMS and PDMS-OH, respectively, and indicate molecular and compositional homogeneity (**Figures S1 and S2**, respectively). The microphase separation of the DBCP is initially justified by differential scanning calorimetry (DSC) results, where two distinctive glass transition temperatures were observed: T_{g1} ~ −115 °C to and T_{g2} ~97 °C, corresponding to non-miscible PDMS and PS segments, respectively (**Figure S3**).

Table S1. Molecular characteristics for PS-*b*-PDMS DBCP.

Sample	$\overline{M}_n^{PS}$ (g/mol) (SEC)	$\overline{M}_n^{PS-b-PDMS}$ (g/mol) (SEC)	I (SEC)	Mass fraction (f_{PS}) (^{1}H-NMR)	Volume fraction (ϕ_{PS}) (^{1}H-NMR)
PS-*b*-PDMS	23,000	38,000	1.06	0.60	0.57

Table S2. Molecular characteristics for PDMS-OH polymer brush.

Sample	(g/mol) (SEC)	I (SEC)
PDMS -OH	5,500	1.06

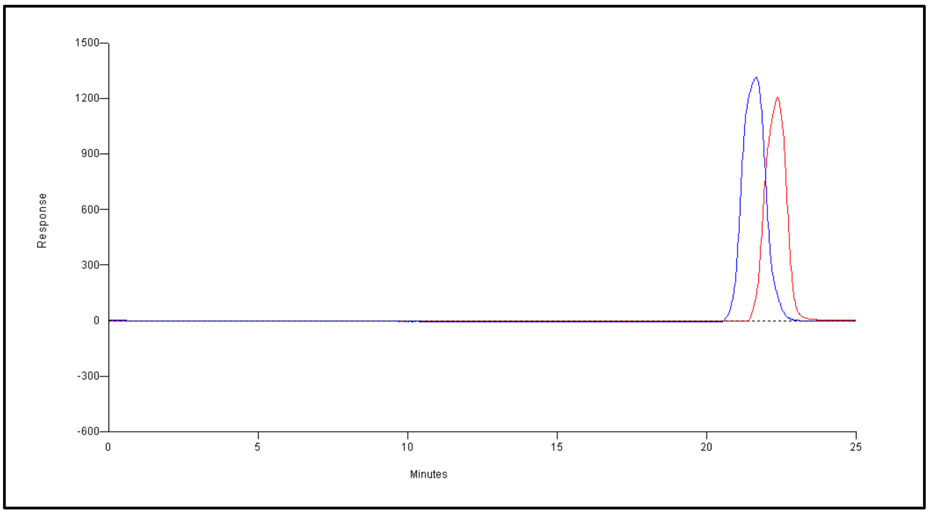

Figure S1. SEC chromatograph of sample PS-*b*-PDMS indicated in blue and the initial precursor PS (indicated in red).

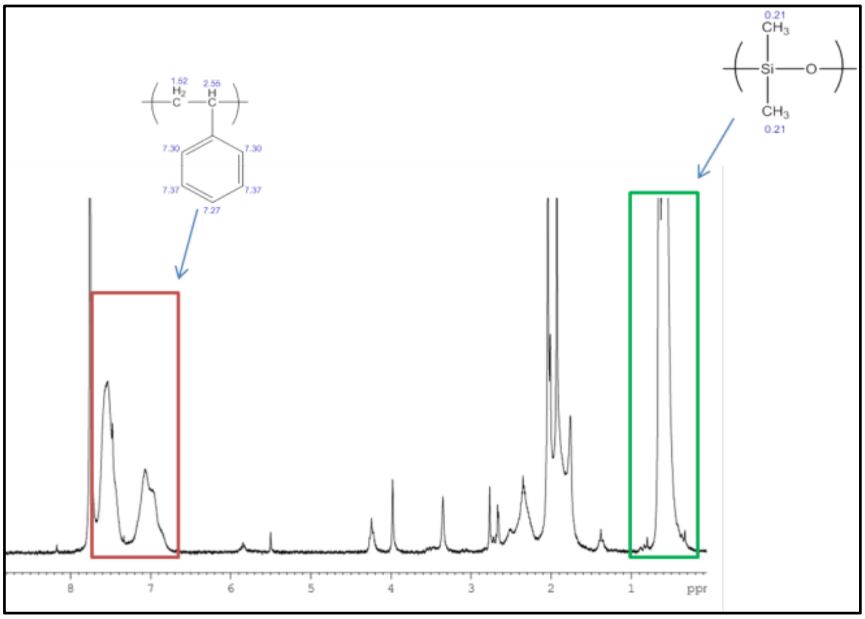

Figure S2. ^{1}H-NMR spectrum of sample PS-*b*-PDMS indicating the chemical shift of one of the six protons for PDMS at ~0.2–0.5 ppm (indicated in green) and the corresponding one of the five aromatic protons for PS at ~6.8–7.5 ppm (indicated in red).

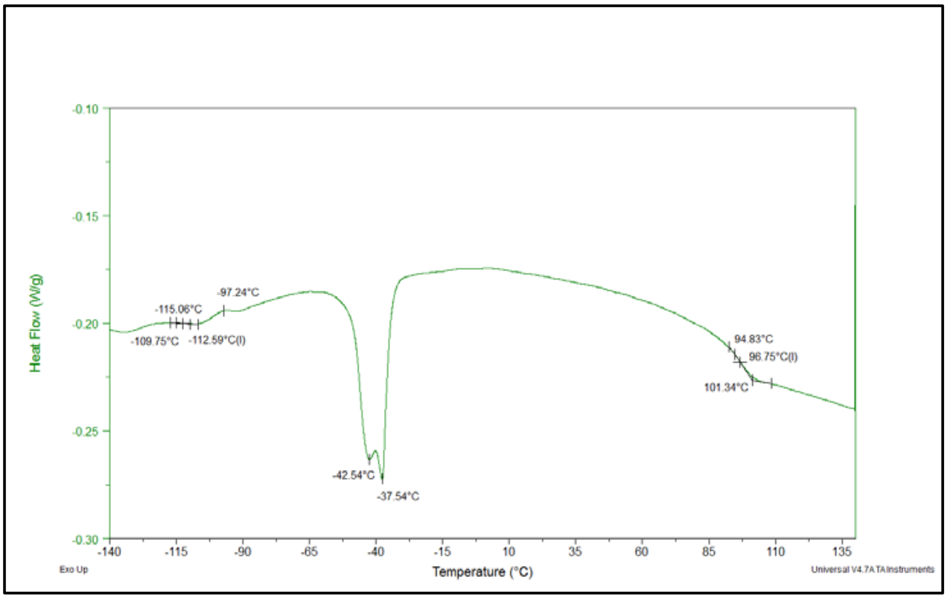

Figure S3. DSC thermograph of sample PS-*b*-PDMS indicating glass transition temperatures of PDMS at ~ −115.06 °C and that of PS at ~96.75 °C.

2. Characterization of Synthesized POSS-C6

2.1. Materials and Methods

2.1.1. Characterization Techniques

^{1}H NMR and ^{13}C NMR spectra were recorded on a 300 MHz Bruker (B-ACS 60) NMR spectrometer in deuterated chloroform ($CDCl_3$). Infrared spectra were performed on a Bruker Tensor 37 spectrometer.

2.2. Results and Discussion

Figure S4 presents the infrared spectra of POSS-C6 obtained by the hydrosilylation of POSS with 1,2-epoxy-5-hexene. The characteristic POSS cage Si–O–Si peak at 1100 cm^{-1} exhibits a similar position and intensity after hydrosilylation, suggesting that the structure survives the processing. In addition to the strong stretching bands of the aliphatic C–H bonds at ~2900 cm^{-1}, a small band is present at 3055 cm^{-1} corresponding to the C–H band in the epoxy ring. The complete functionalization of the POSS cages occurs after 3 hours and can be evidence by infrared spectroscopy by the disappearance of the signal related to the Si–H bond at 2140 cm^{-1} as shown in Figure S5.

^{1}H NMR and ^{13}C NMR spectra of POSS-C6 in Figures S6 and S7 confirm the functionalization of the POSS cage by 8 epoxide ligands, and no another species are present. Moreover, the total disappearance of the ^{1}H NMR peak at 4.2 ppm corresponding to Si–H bond confirms the complete functionalization of the POSS cage. The ^{29}Si NMR spectrum (Figure S8) shows 2 peaks, one at δ 12.71 ppm corresponding to the M-type silicon with the two methyl groups and the alkyl moiety and a second one at δ -108.86 ppm corresponding to the Q-type silicon of the POSS core. No cage cleavage occurs and the POSS core is still intact.

POSS-C6 IR (cm^{-1}): 915, 863 and 837 (ν_{as} epoxy ring), 1100 (ν_s Si–O–Si, POSS), 1250 (ν_s epoxy ring), 1400–1500 (ν_s C–C), 2140 (ν_s Si–H, not present), 2850–2960 (ν_s CH_2–CH_2), 3055 (ν_s CH in epoxy ring)

POSS-C6 ^{1}H NMR ($CDCl_3$, ppm): 0.05 (1: Si–**CH_3**), 0.54 (2: Si–**CH_2**), 1.3–1.5 (3, 4, 5: **CH_2–CH_2–CH_2**), 2.38 and 2.68 (7, 7′: O–**CH_2**–CH epoxy), 2.82 (6: O–CH_2–**CH** epoxy).

POSS-C6 ^{13}C NMR ($CDCl_3$, ppm): 0.0 (1: Si–**C**H_3), 17.9 (2: Si–**C**H_2), 23.1 (3: Si–CH_2–**C**H_2), 29.8 (4: Si–CH_2–CH_2–**C**H_2), 32.5 (5: Si–CH_2–CH_2–CH_2–**C**H_2), 47.4 (7: O–**C**H_2–CH epoxy), 52.5 (6: O–CH_2–**C**H epoxy).

POSS-C6 ^{29}Si NMR (CDCl3, ppm): -100.86 (Q-type Si, POSS core), 12.71 (M-type Si, Ep ligands)

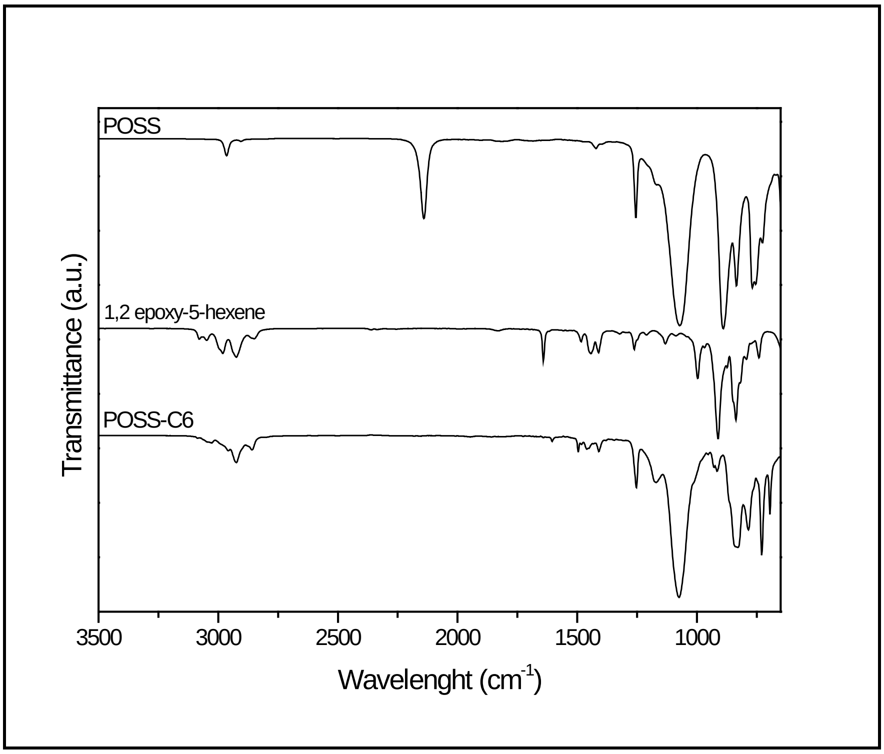

Figure S4. FTIR spectra for POSS, 1,2-epoxy-5-hexene and POSS-C6.

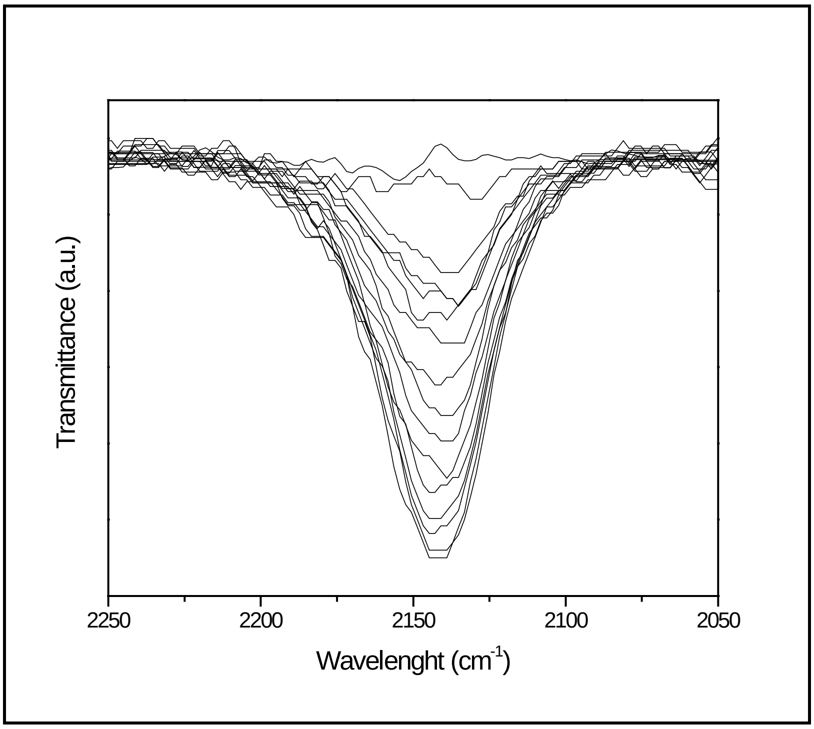

Figure S5. FTIR spectra for POSS-C6 in the Si–H region during the hydrosilylation process (one spectrum every 15 min from down to top).

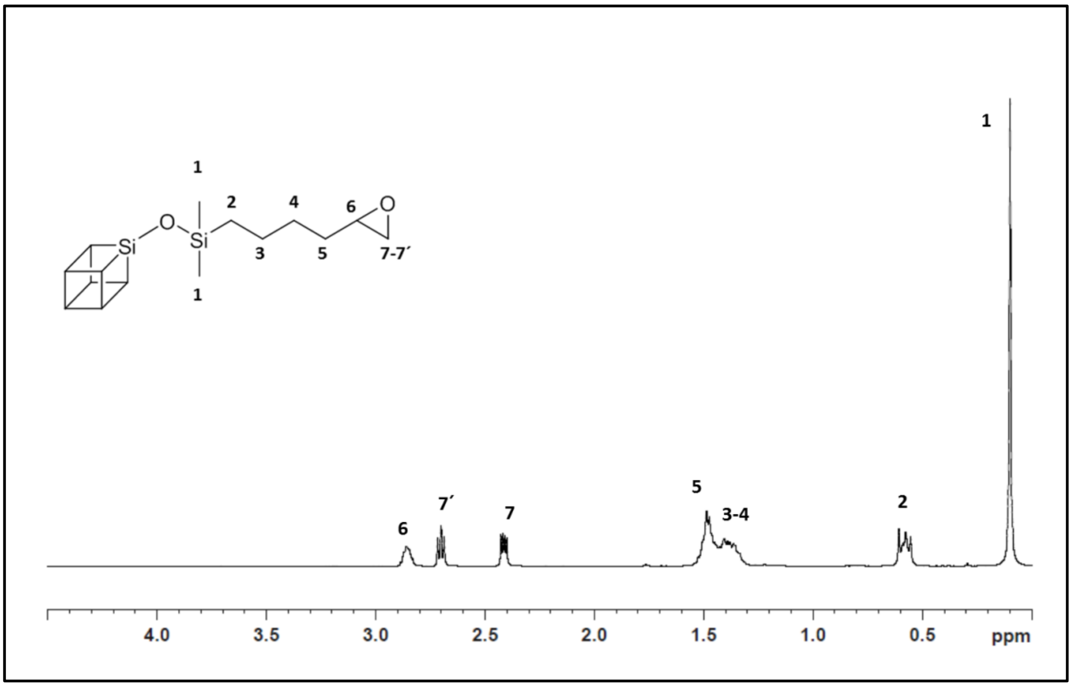

Figure S6. ^{1}H NMR spectrum for POSS-C6

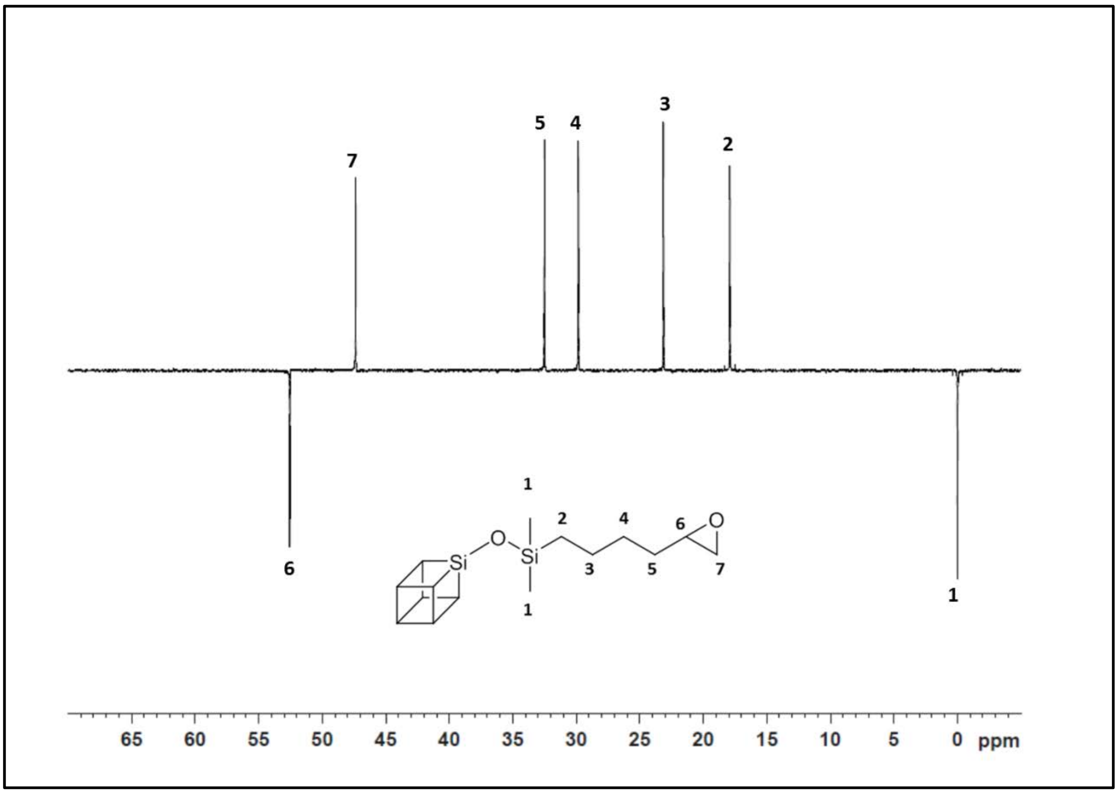

Figure S7. ^{13}C NMR spectrum for POSS-C6.

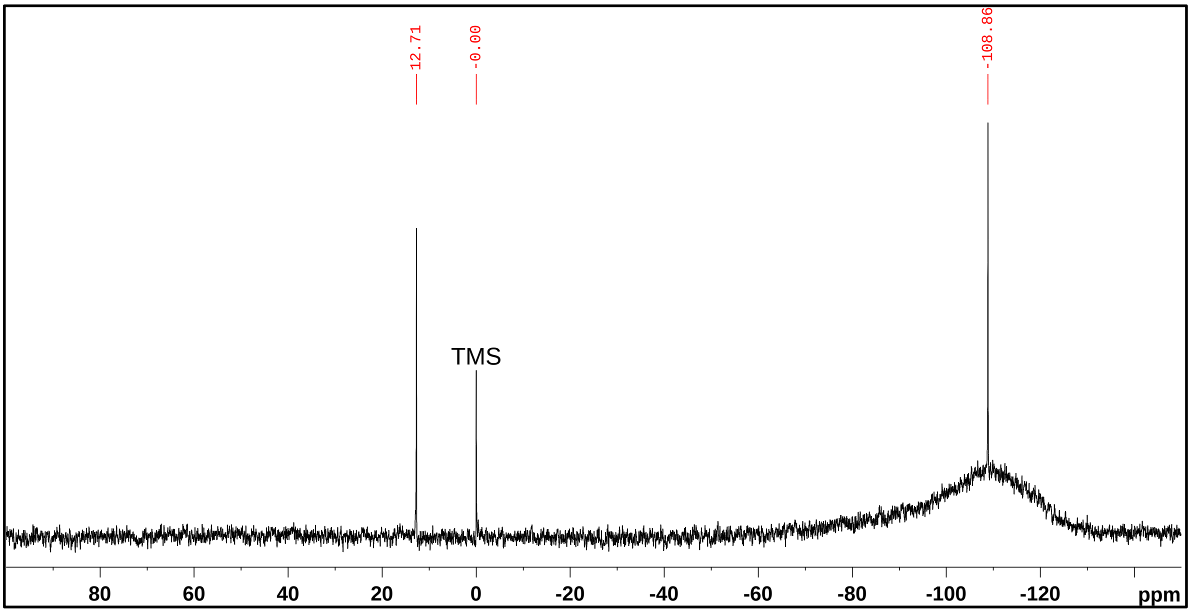

Figure S8. ^{29}Si NMR spectrum for POSS-C6.

References

1. Uhrig, D.; Mays, J.W. Experimental techniques in high-vacuum anionic polymerization. *J. Polym. Sci. Pol. Chem.* **2005**, *43*, 6179–6222.
2. Politakos, N.; Ntoukas, E.; Avgeropoulos, A.; Krikorian, V.; Pate, B.D.; Thomas, E.L.; Hill, R.M. Strongly segregated cubic microdomain morphology consistent with the double gyroid phase in high molecular weight diblock copolymers of polystyrene and poly(dimethylsiloxane). *J. Polym. Sci. Pol. Phys.* **2009**, *47*, 2419–2427.
3. Chao, C.-C.; Ho, R.-M.; Georgopanos, P.; Avgeropoulos, A; Thomas, E.L. Silicon oxy carbide nanorings from polystyrene-b-polydimethylsiloxane diblock copolymer thin films. *Soft Matter* **2010**, *6*, 3582–3587.
4. Chao, C.-C.; Wang, T.-C; Ho, R.-M.; Georgopanos, P.; Avgeropoulos, A; Thomas, E.L. Robust Block Copolymer Mask for Nanopatterning Polymer Films. *ACS Nano* **2010**, *4*, 2088–2094.
